# Increased transcriptome variation and localised DNA methylation changes in oocytes from aged mice revealed by parallel single‐cell analysis

**DOI:** 10.1111/acel.13278

**Published:** 2020-11-17

**Authors:** Juan Castillo‐Fernandez, Erika Herrera‐Puerta, Hannah Demond, Stephen J. Clark, Courtney W. Hanna, Myriam Hemberger, Gavin Kelsey

**Affiliations:** ^1^ Epigenetics Programme Babraham Institute Cambridge UK; ^2^ Science and Biotechnology Faculty, Biology Program CES University Medellin Colombia; ^3^ Centre for Trophoblast Research University of Cambridge Cambridge UK; ^4^ Departments of Biochemistry & Molecular Biology and Medical Genetics Cumming School of Medicine University of Calgary Calgary AL Canada; ^5^ Alberta Children’s Hospital Research Institute University of Calgary Calgary AL Canada

**Keywords:** advanced maternal age, chromatin, DNA methylation, epigenetics, gene expression, oocytes, single‐cell genomics

## Abstract

Advancing maternal age causes a progressive reduction in fertility. The decline in developmental competence of the oocyte with age is likely to be a consequence of multiple contributory factors. Loss of epigenetic quality of the oocyte could impair early developmental events or programme adverse outcomes in offspring that manifest only later in life. Here, we undertake joint profiling of the transcriptome and DNA methylome of individual oocytes from reproductively young and old mice undergoing natural ovulation. We find reduced complexity as well as increased variance in the transcriptome of oocytes from aged females. This transcriptome heterogeneity is reflected in the identification of discrete sub‐populations. Oocytes with a transcriptome characteristic of immature chromatin configuration (NSN) clustered into two groups: one with reduced developmental competence, as indicated by lower expression of maternal effect genes, and one with a young‐like transcriptome. Oocytes from older females had on average reduced CpG methylation, but the characteristic bimodal methylation landscape of the oocyte was preserved. Germline differentially methylated regions of imprinted genes were appropriately methylated irrespective of age. For the majority of differentially expressed transcripts, the absence of correlated methylation changes suggests a post‐transcriptional basis for most age‐related effects on the transcriptome. However, we did find differences in gene body methylation at which there were corresponding changes in gene expression, indicating age‐related effects on transcription that translate into methylation differences. Interestingly, oocytes varied in expression and methylation of these genes, which could contribute to variable competence of oocytes or penetrance of maternal age‐related phenotypes in offspring.

## Graphical abstract

Early embryo development is heavily supported by maternal transcripts and epigenetic states produced in the oocyte. To investigate the effects of age, we compare the transcriptome and methylome of germinal vesicle oocytes from young and old mice using parallel single‐cell RNA sequencing and whole‐genome bisulphite sequencing. We find increased variance in the transcriptome of oocytes from aged females, altered expression of maternal effect genes and differences in gene body methylation at which there were corresponding changes in gene expression.

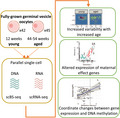

## 1 INTRODUCTION

Over the past 50 years, the proportion of women delaying childbirth in Western societies has steadily increased (Schmidt et al., 2012). Age is the most important factor predicting female fertility potential, with the consequences of advanced maternal age ranging from karyotypic defects in oocytes, leading to aneuploidies and early miscarriage, to defects in placentation arising from compromised decidualisation (Hassold & Chiu, 1985; Lopes et al., 2009; Woods et al., 2017). Within the ovary, age affects both the number and quality of oocytes available for fertilisation (Vollenhoven & Hunt, 2018). Female mammals are born with a fixed pool of oocytes to draw from during their reproductive lifespan. At birth, oocytes are arrested in the ovary at meiotic prophase I, where they will remain quiescent for months or years, depending on the species. Only a small subset will ever fully mature and ovulate, as the vast majority will undergo atresia before reaching ovulation. During the long quiescent phase, molecular changes in the oocyte result in a decline in oocyte developmental competence with advancing maternal age. Amongst the key molecular changes found to occur in oocytes from aged females are mitochondrial dysfunction, telomere shortening, cohesin dysfunction resulting in spindle instability and reduced stringency in spindle assembly checkpoints (Reviewed in Cimadomo et al., 2018; Vollenhoven & Hunt, 2018). In recent years, it has been proposed that transcriptional and epigenetic changes, especially DNA methylation, may contribute to compromised oocyte quality with advanced maternal age (Cimadomo et al., 2018; Ge et al., 2015; Marshall & Rivera, 2018). The changing environment to which an oocyte is exposed in the ageing ovary, such as altered hormone levels, and changes in energy and one‐carbon metabolism, could affect gene expression and epigenetic processes that could contribute to the lower developmental capacity of oocytes with age (Ge et al., 2015). Effects on epigenetic quality of the oocyte are also important because they can potentially be inherited into the embryo, manifesting as later developmental outcomes in offspring.

Gene expression in the oocyte is unique in that it comprises many oocyte‐specific transcripts that not only regulate processes in the oocyte, but also in early embryo development. The growing oocyte accumulates and stores transcripts until it undergoes transcriptional arrest prior to ovulation. At this stage, transcription ceases until after fertilisation when it resumes during zygotic genome activation (Reviewed in Svoboda et al., 2015; Winata & Korzh, 2018). This means that all processes occurring during oocyte maturation, fertilisation and zygotic genome activation are regulated by the previously stored maternal transcripts and proteins, and changes in transcription or transcript storage capacity during ageing could therefore influence the developmental potential of the oocyte. Whole‐transcriptome analysis using expression microarrays in both human and mouse metaphase‐II (MII) oocytes has shown that typically a few hundred transcripts have altered abundance in the ageing oocyte and has identified genes involved in cell‐cycle regulation, spindle assembly, oxidative stress and DNA damage as being frequently affected (Barragán et al., 2017; Grøndahl et al., 2010; Hamatani et al., 2004; Pan et al., 2008; Steuerwald et al., 2007). More recent studies using single‐cell RNA‐seq in human MII oocytes have found similar results, although the number of differentially abundant transcripts detected varied widely between studies (Barone et al., 2020; J.‐J. Zhang et al., 2020).

Besides providing maternal factors for oocyte competence, transcription in the oocyte is also required for determining where *de novo* DNA methylation occurs in the genome. In the female germline, DNA methylation is globally reprogrammed during the course of gametogenesis. DNA methylation is almost completely erased in the primordial germ cells during embryonic development (Gkountela et al., 2015; Guibert et al., 2012; F. Guo et al., 2015; H. Guo et al., 2017; Seisenberger et al., 2012), after which the genome remains essentially unmethylated until *de novo* methylation initiates in the later stages of oocyte growth in the postnatal ovary (Gahurova et al., 2017; Hiura et al., 2006). *De novo* methylation during oocyte growth is an intricately regulated process that could be influenced both by internal and external factors, and changes in oocytes with age could influence the ultimate DNA methylation pattern established. For example, changes in nutrient supply and one‐carbon metabolism could reduce the amount of substrate available for DNA methyltransferases (DNMTs) (Ge et al., 2015). In mouse oocytes, methylation is catalysed by DNMT3A together with its co‐factor DNMT3L (Bourc'his, 2001; Kaneda et al., 2004) and is targeted to regions of active transcription, resulting in a bimodal distribution, unique to the oocyte, of broad unmethylated domains and methylated regions overlapping transcribed genes (Kobayashi et al., 2012; Veselovska et al., 2015). This link between transcription and DNA methylation predicts that transcriptional changes in oocytes with age could affect DNA methylation by changing recruitment of DNMTs to chromatin and/or by altering expression levels of DNMTs and related proteins. Decreased expression of DNMT3A, DNMT3L as well as the maintenance methyltransferase DNMT1, has been reported in ageing mouse oocytes, both at the transcript and protein level (Hamatani et al., 2004; Yue et al., 2012). Whether these changes in DNMT expression are sufficient to affect DNA methylation levels has so far only been assessed on a global level using immunofluorescence (IF): Yue et al. found that the decrease in DNMT protein abundance correlated with a small but significant decline in global DNA methylation levels in MII oocytes and preimplantation embryos (Yue et al., 2012).

Successful epigenetic reprogramming is essential for the developmental capacity of the oocyte, as genomic imprinting is established in this phase. Genomic imprinting, defined as parent‐of‐origin‐dependent gene expression in offspring conferred by differential methylation inherited from the gametes, is the clearest demonstration of long‐term intergenerational epigenetic inheritance (Ferguson‐Smith, 2011). DNA methylation established in oocytes also contributes to proper development of the trophoblast lineage (Branco et al., 2016). Imprinting has been reported to be stable in embryos and placentas from ageing mice, indicating correct establishment in oocytes and maintenance in the early embryo (Lopes et al., 2009). Consistent with this earlier report, recent analysis of the germline differentially methylated regions (gDMRs) that control the imprinted genes *Snrpn*, *Kcnq1ot1* and *H19 *has shown correct levels of DNA methylation in oocytes irrespective of advanced maternal age, as well as normal methylation maintenance during preimplantation development (Kindsfather et al., 2019). On the other hand, as we recently showed, there are localised and coherent differences in DNA methylation in oocytes collected from prepubertal female compared with young adult mice, indicating that the DNA methylation landscape of the oocyte is not a completely fixed entity but is modulated during the life‐course (Saenz‐De‐Juano et al., 2019). However, until now, no quantitative genome‐wide assessment of methylation at single‐base resolution in oocytes from aged females has been done.

Since ovaries from reproductively old mice contain fewer follicles, the number of oocytes in which to study gene expression and DNA methylation at a genome‐wide level is severely limited. Such limitations have been overcome by recent advances in single‐cell sequencing technologies (Kelsey et al., 2017). Here, using the single‐cell M&T‐seq approach (Angermueller et al., 2016), which allows the physical separation of RNA and DNA from single cells, we set out to generate RNA‐sequencing (scRNA‐seq) and whole‐genome bisulphite sequencing (scBS‐seq) libraries from the same oocytes from young (12 weeks) and old (>40 weeks) mice. The profiled cells were fully grown germinal vesicle (GV) oocytes, which are already transcriptionally silent and have acquired most of the DNA methylation of mature oocytes including genomic imprinting marks. In accordance with previous studies, we identified transcript abundance changes in oocytes from aged compared to young mice affecting a relatively small number of genes. However, because of the single‐cell resolution, we were uniquely able to identify increased molecular heterogeneity amongst older oocytes. While the majority of aged oocytes showed features suggestive of reduced developmental competence, a few retained a ‘young‐like’ profile. Similar to the transcriptome, the methylome was globally similar irrespective of age, although specific changes in DNA methylation between oocytes from aged and young females were identified. Furthermore, because of the parallel profiling, we were able to link some transcript changes to methylation changes in the same oocyte, indicating that loss of gene expression fidelity in the aged oocyte can result in altered DNA methylation.

## 2 RESULTS

### 2.1 Global patterns of gene expression in oocytes are associated with age and chromatin configuration

**Figure 1:**
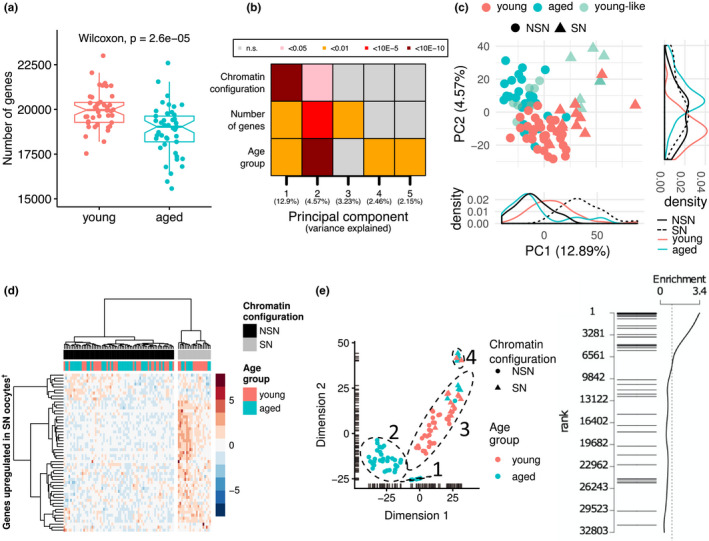
Transcriptomic profiles of oocytes from young and old females. (a) Boxplots of transcript diversity showing a decrease in oocytes from aged mice (Wilcoxon test;*p* = 2.6x10^−5^). Each dot represents the number of genes detected at ≥1 counts in scRNA‐seq of an individual MII oocyte. (b) Heatmap of the associations (linear regression) between a transcriptional signature of chromatin configuration, number of detected transcripts and age with the first five principal components of the transcriptome. (c) Principal component analysis (PCA) plot of the transcriptome of oocytes from young and aged mice. Principal component 1 (PC1) is highly explained by a predicted chromatin configuration (non‐surrounded nucleolus (NSN): circles; surrounded nucleolus (SN): triangles). PC2 is highly explained by age (young: red; aged:turquoise). A subpopulation of aged oocytes with young‐like (light turquoise) features is also demarcated. (d) Hierarchical clustering for the prediction of chromatin states of oocytes as NSN (black) or SN (grey) according to the level of expression of genes overexpressed in SN oocytes. (e) t‐SNE plot showing four main clusters of oocytes driven by inferred chromatin state and/or age group. (f) Barcode plot showing the enrichment of maternal effect genes when testing for differences across the four main clusters of oocytes. Each horizontal bar represents one maternal effect gene. The position of the bar along the*y*‐axis represents its ranking across all expressed genes tested for differential expression (1 being the most significant)

In order to investigate the effect of maternal age on global gene expression and DNA methylation in oocytes, we applied scM&T‐seq (Angermueller et al., 2016) for joint transcriptome and methylome profiling from the same individual oocytes. Fully grown GV oocytes were isolated from the ovaries of three reproductively young, virgin female mice (C57BL/6Babr, 12 weeks old) and from seven reproductively old, virgin females (44–54 weeks old), an age at which females of this strain are approaching the end of their reproductive lifespan (Woods et al., 2017). scRNA‐seq libraries that passed quality control filtering were obtained from 42 young and 45 aged oocytes. On average, each oocyte contained transcripts from 19,400 genes (Table S1), from which 11,578 were shared across all oocytes. However, a lower number of expressed genes was observed in the aged group (difference in means=1,147 genes; Wilcoxon test, *p* = 4.2 × 10^−5^; Figure 1A), indicating a reduced complexity in the transcriptome of GV oocytes from aged females.

To further explore the effect of age on global patterns of gene expression, we used principal component analysis (PCA) as implemented by the denoisePCA method in Scran (Lun et al., 2016). This programme partitions the total variance into its technical and biological components assuming that random noise is the major contributor to the variance of most genes in a scRNA‐seq data set, but is uncorrelated across genes and should only be captured by later principal components (PCs) (Lun et al., 2016). In our data set, approximately 25% of the variance, which explained the first five PCs, was attributed to biological components. Age was identified as an important contributor to global variation in transcript abundance as it was associated with PCs 1, 2, 4 and 5, with PC2 being the most strongly associated with age (*p* = 1.01 × 10^−12^) (Figure 1b). PC1 as the main axis of variation was explained in part by age, although not as strongly as PC2, which suggested another important source of variation together with age. By comparing the loadings on PC1 between the age groups, we observed that aged oocytes showed a multimodal distribution compared to the unimodal distribution displayed by the young oocytes (Figure 1c), which suggested that the unknown source of variation was driving heterogeneity to a greater extent in the aged group of oocytes.

A key feature in determining maturation and competence of GV oocytes, and that might contribute a source of variation in oocytes at this stage, is chromatin condensation configuration: the transcriptionally active ‘non‐surrounded nucleolus’ (NSN) configuration or the more mature and transcriptionally inactive ‘surrounded nucleolus’ (SN) configuration. This chromatin arrangement dictates developmental competence as fertilised oocytes with a NSN configuration mostly arrest at the two‐cell stage (Monti et al., 2013). To assign chromatin configuration states in our data set, we classified the 87 oocytes according to the level of expression of genes reported to show at least a two‐fold overexpression in SN oocytes compared to NSN oocytes (Ma et al., 2013). Twenty oocytes were found to express these transcripts at a higher level and were classified transcriptionally as SN (Figure 1D). A very strong association between the predicted chromatin configuration and PC1 was observed (*p* = 4.03 × 10^−18^), which suggested that the main source of variation in the data set was a transcriptional profile of chromatin configuration linked to developmental competence (Figure 1b,c).

To assess whether further comparisons between age groups would be confounded by chromatin configuration, we tested for a difference in proportions in assigned chromatin configurations between the two age groups. This assessment was important as it has been reported that the SN:NSN ratio amongst GV oocytes increases with age (Zuccotti et al., 1995). Out of the 20 SN oocytes, twelve corresponded to the young group and eight to the aged one. The proportion of transcriptionally‐like SN oocytes was not significantly different between groups (Fisher's exact test, *p* = 0.46), indicating that our populations are comparable for this parameter, and any observed age effects would not be confounded by oocyte developmental stage. However, we also observed that the number of SN oocytes was low compared to the numbers reported in the literature, especially in our aged group in which more than 80% of oocytes are expected to be SN. It is likely that the classification obtained using gene expression as a proxy does not reflect the actual chromatin state, but instead suggests that most aged oocytes expected to be SN express an immature NSN‐like transcriptome.

### 2.2 Unsupervised clustering reveals both age‐ and chromatin configuration‐driven oocyte populations with differential expression of maternal effect genes

Dimensionality reduction by t‐distributed Stochastic Neighbor Embedding (t‐SNE) revealed four well‐separated clusters of oocytes in our data set (Figure 1e). Clusters 1 and 2 comprised only NSN aged oocytes; cluster 3 mainly comprised young NSN oocytes plus a small number of SN oocytes; and cluster 4 purely SN oocytes regardless of age, which were closer to young NSN oocytes (cluster 3) than to aged NSN oocytes (clusters 1 and 2). This spatial relationship suggested a trajectory from an immature NSN transcriptome (cluster 1) to a mature SN one (cluster 4). Following this assumption, differential expression was tested using cluster number as a continuous variable to identify transcripts that change in abundance from old transcriptionally‐like NSN oocytes to young transcriptionally‐like NSN oocytes and, lastly, to SN oocytes (both young and aged). In total, 6,464 transcripts were identified as differentially abundant (Bonferroni adjusted *p* < 0.05; Table S2). Interestingly, the top ranked transcripts in this analysis were enriched for maternal effect genes (e.g., *Padi6*, *Nlrp5*, *Gdf9*, *Tcl1*, *Dnmt1*, *Uhrf1*, *Bmp15* and *Dppa3*) (Figure 1F and Table S2). To exclude that the observed differences in transcript abundance of maternal effect genes were an effect solely of chromatin configuration, the analysis was repeated using only the subset of oocytes assigned as NSN and the enrichment for maternal effect genes was also observed (Figure S1).

### 2.3 Aged oocytes exhibit greater gene expression variability

**Figure 2:**
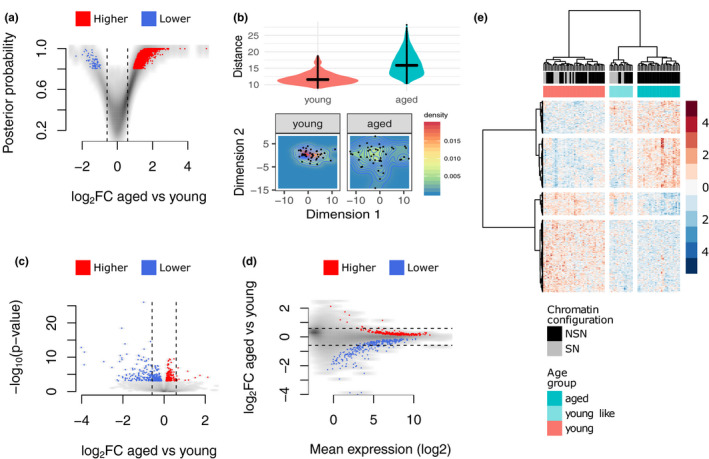
Differences in variability and mean levels of transcript abundance. (a) Volcano plot of differential variability between young and aged oocytes showing a greater number of genes having more variable expression values in the aged group. (b) Upper: boxplot of the pairwise distances between cells of the same age group. Lower: heatmap of the 2D distribution of oocytes using the expression level of genes identified as differentially variable between young and aged oocytes. (c) Volcano plot of differential mean expression between young and aged oocytes. (d) Scatter plot of the effect size vs. mean expression level of genes tested for differential expression. (e) Hierarchical clustering of oocytes using expression levels of 560 age‐associated genes showing a young‐like subgroup within the aged oocytes. In A, C, D, red and blue dots represent higher or lower variability (A) and mean expression (C, D), respectively, in the aged group

Increased heterogeneity in gene expression with age was suggested by the different distributions of the two age groups along the PC1 axis (Figure 1C). To assess the increased variability at the gene level rather than at global patterns, and to identify those genes becoming variable with age, we tested for differential variability in gene expression between young and aged oocytes. A total of 2,811 genes showed higher expression dispersion in aged oocytes, while only 74 decreased in variability compared to the young group, which suggests a more stochastic gene expression profile with increased age (Figure 2A and Table S3). We then estimated the pairwise distances between all oocytes in each group at these differentially variable regions, which indicated that cell profiles were further away from each other in the aged group (Figure 2b).

### 2.4 A fraction of aged oocytes expresses a young‐like transcriptome

In addition to differences in variability, we also tested for differences in mean expression levels. We identified differences between young and aged oocytes at 560 transcripts (FDR <0.05; Figure 2c and Table S4), of which 156 showed a fold change ≥1.5. Out of the 560 differentially expressed genes (DEGs; note we use this term without assuming a transcriptional basis as post‐transcriptional effects may also influence transcript abundance), 300 (53.6%) showed a reduction in abundance in the aged oocytes, while 260 a gain. DEGs were observed across the whole range of expression; however, low count genes were mostly down‐regulated in aged oocytes, while highly abundant transcripts were seen to be both down‐ and up‐regulated (Figure 2d). No significantly enriched gene ontology terms were observed. Hierarchical clustering based on these 560 DEGs identified a population of aged oocytes (*n* = 16), which we refer to as the young‐like group, that expressed a subset of 172 transcripts at levels similar to those observed in the young group (Figure 2e and Table S5). There were no significantly enriched gene ontology terms in this subset of transcripts. The young‐like subpopulation within the aged oocytes might account for the higher variability observed in the transcriptomes of the aged group (Figure 1c and Figure 2A,B). Interestingly, all of the predicted SN aged oocytes were also defined as young‐like oocytes, although the young‐like group also contained some aged oocytes assigned as NSN (Figure 2e).

### 2.5 Reduced global DNA methylation but preserved methylation landscape in aged oocytes

**Figure 3:**
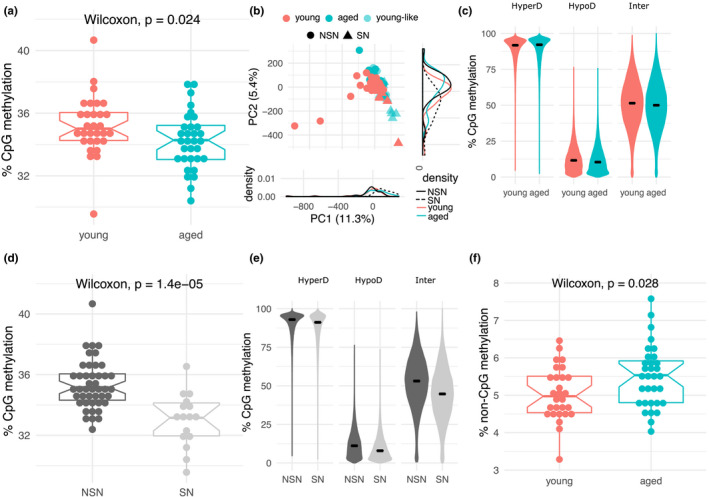
DNA methylation profiles of individual oocytes from young and aged females. (a) Boxplot of average CpG methylation values showing lower levels in aged oocytes (Wilcoxon test;*p* = 0.024). Each dot represents the global CpG methylation estimate from scBS‐seq of 32 individual GV oocytes from young and 30 from aged females. (b) PCA plot of oocyte CpG methylomes of young and aged oocytes based on the average methylation at hyper‐, hypo‐ and intermediately methylated domains. (c) Violin plots of the distribution of average CpG methylation values across three types of methylation domains in the oocyte: hypermethylated (HyperD: 75‐100%); hypomethylated (HypoD: 0‐25%) and intermediately methylated (Inter: 25‐75%) in young and aged oocytes. scBS‐seq data merged by group. (d) Boxplot of average CpG methylation values in oocytes assigned as NSN and SN (Wilcoxon test;*p* = 1.4x10^−5^). (e) Violin plots of the distribution of average CpG methylation values of HyperD, HypoD and Inter domains in oocytes assigned as NSN and SN. scBS‐seq data merged by group. (F) Boxplot of average non‐CpG methylation values in young and aged oocytes (Wilcoxon test;*p* = 0.028)

A tight relationship between gene expression and DNA methylation has been identified in the oocyte, in which 85–90% of DNA methylation can be attributed to transcriptional events (Veselovska et al., 2015). In order to detect possible coordinate changes between gene expression and DNA methylation, the same GV oocytes from which scRNA‐seq libraries were obtained were processed for scBS‐seq, from which 30 oocytes from the young group and 32 oocytes from the old group yielded libraries that passed quality control filtering (Table S6). These scBS‐seq libraries yielded a mean coverage of 3,203,801 CpGs (743,802‐6,536,709), equivalent to 14.7% (3.4‐29.9%) of the genomic CpGs that are accessible by bisulphite sequencing. Global levels of CpG methylation were between 29.6‐40.7% (Figure 3a), within expectations for the global levels estimated by scBS‐seq at this degree of sequence coverage (Smallwood et al., 2014). The average methylation level of oocytes from aged females was lower than that of young females (Wilcoxon test; *p* = 0.024; Figure 3a).

Because fully grown mouse oocytes have such a distinctive DNA methylation pattern, with high and uniform methylation over expressed gene bodies and low methylation of intergenic regions, we estimated mean CpG methylation values at hypomethylated, hypermethylated and intermediately methylated domains and then performed PCA to investigate if global patterns are affected by age. Although no clear segregation was observed (Figure 3b), age groups showed a difference in PC2 (Wilcoxon test; *p* = 0.02) and a suggestive difference in PC1 (Wilcoxon test; *p* = 0.07) (Figure S2). We then assessed the distribution of CpG methylation values across the three different genomic categories, but only small changes were observed (Figure 3c), indicating that the DNA methylation landscape is generally preserved. However, when examining differences between assigned chromatin configurations, lower CpG methylation was observed in SN‐classified oocytes both globally (Wilcoxon test; *p* = 1.4x10^−5^) and in all three categories of genomic features (Figure 3d,e). This finding suggests that transcriptional status and/or developmental competence are more important factors in the ultimate methylation pattern than age itself. Oocytes are unusual in accumulating higher levels of non‐CpG methylation (Shirane et al., 2013; Tomizawa et al., 2011). In contrast to the effect observed for CpG methylation, oocytes from aged females had increased non‐CpG methylation levels (Wilcoxon test; *p* = 0.028; Figure 3f).

**Figure 4:**
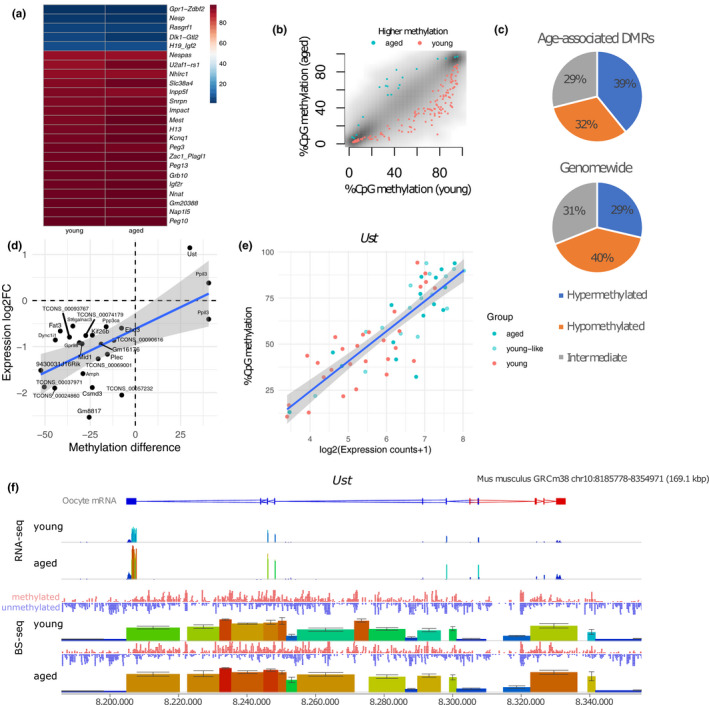
Localised DNA methylation changes in oocytes from aged females. (a) Heatmap showing methylation levels at 19 maternal gDMRs, three paternal gDMRs (*Rasgrf1*,*Dlk1*‐*Gtl2*,*H19_Igf2*) and secondary DMRs (*Nesp*and*Gpr1*/*Zdbf2*). Shown are the CpG methylation levels of each DMR merged across the 30 young or 32 aged oocyte scBS‐seq data sets. (b) Scatter plot showing the average methylation in aged vs. young oocytes of high‐confidence DMRs (hyper‐ (HyperD), hypo‐ (HypoD) and intermediately methylated (Inter) domains) with higher levels in aged or young oocytes marked in turquoiseor red, respectively. DMRs were defined as significant at a false discovery rate of 5% in at least 95% of 100 pseudobulk combinations of 10 oocytes. (c) Pie chart of the percentage of high‐confidence DMRs within hyper‐ (HyperD), hypo‐ (HypoD) and intermediately methylated (Inter) domains and the expected genomic distribution. (d) Coordinate changes in gene expression and CpG methylation at 23 genes in aged oocytes showing a positive association. Methylation difference refers to difference in % CpG methylation in merged data of young and old oocytes. (e) Scatter plot of*Ust*as an example of coupled changes between gene expression and CpG methylation at single‐cell level. (f) Seqmonk genome browser of a 169.1 kbp interval surrounding the*Ust*locus. The RNA‐seq tracks quantify counts at 2 bp windows. In the BS‐seq tracks, each vertical block represents a hyper‐, hypo‐ or intermediately methylated domain. Height and colour‐coding indicate read counts and % methylation, respectively. Methylation values of 100‐CpG tiles represent mean of three pseudobulk groups each comprising 10 scBS‐seq data sets, with standard error indicated. Tracks labelled methylated CpG (red) and unmethylated CpG (blue) indicate total reads at individual CpGs for the scBS‐seq data sets merged by young and aged groups. On the top, the track labelled oocyte mRNA depicts exonic structures of oocyte transcripts with red indicating transcription from left to right and blue indicating transcription from right to left

Amongst the functionally most significant sites of DNA methylation in the oocyte are the gDMRs that control imprinting. Maternal gDMRs need to acquire methylation fully in oocytes to ensure imprinted monoallelic expression of imprinted genes in the embryo after fertilisation, and most imprinted genes are controlled by gDMRs methylated in oocytes (Schulz et al., 2010). We found that the 19 maternal gDMRs we assessed were highly methylated in oocytes irrespective of maternal age; in addition, three paternal gDMRs and two secondary DMRs were unmethylated similarly in both age groups (Figure 4a). Moreover, there was no evidence of increased variability in gDMR methylation in oocytes from aged females when analysed individually (Figure S3).

### 2.6 Age‐associated differentially methylated regions

In addition to a global reduction in CpG methylation detected between the oocytes from young and old females, we wished to ascertain whether there were discrete or recurrent age‐associated methylation differences. To do so, and to mitigate against sub‐genome coverage of single oocyte data sets, we created randomised pseudobulk groups of 10 single oocyte data sets from the young and aged groups. Further, we generated 100 different combinations/permutations of the pseudobulk groups in order to limit the rate of false‐positive signals that could arise from the composition of the groups (e.g., differences in coverage). Signals that appeared as significant at a false discovery rate of 5% in at least 95% of the combinations were carried forward as age‐associated DMRs. By this approach, we identified 166 high‐confidence DMRs across the genome (Figure 4b) of which 65 were within hypermethylated domains, 53 within hypomethylated domains and 48 within intermediately methylated domains (Figure 4c). The percentage of DMRs in hypermethylated domains was higher than expected from genomic proportions, and, conversely, the percentage in hypomethylated domains was lower than expected. Independently of the type of domain, the majority of DMRs (88.0%) showed reduced methylation in the aged oocytes (Figure 4b). We then used an imputation and clustering method for single‐cell methylomes (Kapourani & Sanguinetti, 2019) to validate if these DMRs were able to classify oocytes accurately as young or aged. All 30 young oocytes were correctly classified, and only one of the 32 aged oocytes was mislabelled as young (Table S7).

### 2.7 Coordinate changes between gene expression and DNA methylation at a subset of genes

Because *de novo* methylation in oocytes is strongly associated with gene transcription, we sought to identify the overlap between and DEGs and DMRs to explore the relationship between gene expression and DNA methylation in relation to ageing in the oocyte. In total, 33 of the 166 DMRs overlapped with 23 different genes. For these genes, a positive correlation between the difference in methylation and fold change in gene expression was observed (Figure 4d), indicating that in these cases the DNA methylation differences could be the result of transcriptional differences between young and old oocytes, given that transcription precedes gene body DNA methylation in oocytes as shown by previous studies (Kobayashi et al., 2012; Veselovska et al., 2015). To explore this in more detail, we estimated the correlation coefficient between gene expression and DNA methylation over gene bodies at the single‐cell level and found significant (*p* < 0.05) positive correlations for 14 of them (Figure S4). As shown for the gene *Ust*, individual oocytes varied both in expression and gene body methylation, demonstrating a strong linear correlation (Figure 4e) and resulting in increased DNA methylation of *Ust* in the population of aged oocytes (Figure 4f). At these genes, with one exception, we also found that non‐CpG methylation correlated positively with the level of CpG methylation (Figure S5), consistent with the notion that non‐CpG methylation is also dependent on the activity of DNMT3A and obeys similar genomic targeting principles as CpG methylation (Shirane et al., 2013).

## 3 DISCUSSION

In this study, we have undertaken a parallel, single‐cell assessment of the transcriptome and DNA methylome of fully grown GV oocytes from reproductively young and old female mice in order to understand the impacts of maternal age on the epigenetic properties of the oocyte. The importance of proper transcriptional and epigenetic regulation in the oocyte goes far beyond its competence for maturation and fertilisation; early embryonic development is dependent on it. One of these factors is a pool of transcripts that will be needed prior to zygotic genome activation. Oocytes have unique RNA dynamics: transcripts accumulate over the extended period of oocyte growth as well as being subject to extensive processing upon oocyte maturation. As our transcriptome analysis was based on scRNA‐seq, it is a record of transcript abundance at the time of oocyte collection rather than a direct measure of transcription *per se*. Therefore, many of the differences we detect with age could reflect post‐transcriptional effects. We identified 560 differentially abundant transcripts between GV oocytes from young and old females, and, consistent with a likely post‐transcriptional basis for the majority of these differences, only a small fraction (23 out of 560) had corresponding changes in gene body DNA methylation. Nevertheless, the scRNA‐seq data can be regarded as a reflection of developmental competence of the oocyte. In our scRNA‐seq analysis, we identified two key properties of the transcriptomes of GV oocytes from aged females: a reduced complexity in the number of transcripts and increased variability. It is plausible that as oocytes in ovaries from ageing females remain in an arrested state for a longer period of time, stochastic changes in the transcript pool may account for this observation. It was also striking that not all aged oocytes were affected to the same degree as a fraction of them (the young‐like group) partially resemble the profile of oocytes from young, fertile females. It is only through the application of single‐cell profiling methods that such age‐related heterogeneity can be revealed.

The culmination of oocyte growth is the transition from the transcriptionally active NSN to the inactive SN configuration, in which state the GV oocyte remains until GV breakdown (GVBD) and the resumption of meiosis. We did not determine the chromatin organisation of oocytes on collection, but inferred this by reference to existing RNA‐seq data sets (Ma et al., 2013), thereby assigning oocytes as transcriptionally NSN‐like and SN‐like. NSN and SN oocytes have different developmental potential (Zuccotti et al., 1998), and the associated transcriptome differences are a reflection of this competence (Ma et al., 2013; Monti et al., 2013). We scored a surprisingly high proportion of oocytes from older females as NSN from their transcriptomes at an age when the ovary should contain few NSN oocytes (Zuccotti et al., 1995). On the other hand, it has been reported that more than a quarter of GV oocytes in old female mice cannot be classified as either NSN or SN, but display anomalous chromatin configurations (Manosalva & González, 2010). Together, these observations suggest that aged oocytes may have undergone the NSN‐SN transition, but imperfectly and without the full transcriptome maturation that ensures developmental competence. Consistent with this notion, we find that transcripts distinguishing SN oocytes from the old transcriptionally NSN‐like oocytes were enriched in maternal effect genes that are critical for viability of the early embryo.

DNA methylation globally was similar between oocytes from young and aged females, although there was a slight reduction in total CpG methylation but increase in non‐CpG methylation in the aged group. In addition, the characteristic DNA methylation landscape of alternating hyper‐ and hypomethylated domains was preserved. Consistent with our results, a previous study reported a slight reduction in 5‐methylcytosine in MII oocytes from aged compared with young females as assessed by immunofluorescence (Yue et al., 2012). IF signal is likely to report predominantly on repetitive DNA sequences such as pericentromeric heterochromatin, a genomic fraction that BS‐seq is less able to quantify owing to read mapping and methylation calling ambiguities of repetitive sequences. *De novo* methylation is progressive over oocyte growth, so a reduction in total CpG methylation could indicate slightly attenuated activity or fidelity of the *de novo* methylation complex in oocytes from aged females: quite substantial reductions in DNMT3A and DNMT3L protein levels in aged oocytes have been reported (Yue et al., 2012). Alternatively, there could be elevated turnover of DNA methylation with age, although active demethylation is not known to occur in oocytes. A surprising observation was the reduced CpG methylation in oocytes assigned as SN‐like.

Much of the DNA methylation the oocyte accumulates during its growth is of unknown functional significance, as oocytes deficient in DNMT3L or DNMT3A and therefore lacking most DNA methylation are able to be ovulated and fertilised with embryo progression until early post‐implantation stages (Bourc'his, 2001; Kaneda et al., 2004). Functionally, the most important methylation in oocytes occurs at gDMRs of imprinted genes. Our results show that *de novo* methylation of imprinted gDMRs in aged oocytes is not impaired. This accords with a recent study of the *Snrpn* and *Kcnq1ot1* gDMRs in mouse oocytes that reported normal methylation acquisition irrespective of age, as well as no effect on maintenance of methylation to the blastocyst stage following fertilisation of oocytes from aged females (Kindsfather et al., 2019). Methylation in embryos and placentas at embryonic day 10.5 of aged mice was previously found to be normal at both maternal and paternal gDMRs (*Snrpn*, *U2af1*‐*rs1*, *Peg1*, *Igf2r*, *H19*) (Lopes et al. 2009); methylation of the *SNPRN* gDMR is also reported to be unaffected by age in bovine oocytes (Mattern et al., 2016).

By using a very stringent approach that accounts for the relatively sparse and random coverage of scBS‐seq data sets, we were able to identify 166 high‐confidence age‐related DMRs, the majority of which showed less methylation in oocytes from aged females. DNA methylation in the oocyte is tightly linked to transcription events (Kobayashi et al., 2012; Veselovska et al., 2015), and for 33 of these DMRs we find concordant changes in transcript and methylation level, indicating that there are likely to be *bona fide* age‐related differences in transcription that feed forward into differences in DNA methylation for this subset of genes. For some of these genes, both transcript abundance and DNA methylation varied within the age groups, indicating heterogeneity in expression amongst oocytes. Although many of these genes have no established role in oocyte development or function, there are some interesting cases. For example, *Dync1i1* encodes a component of the cytoplasmic dynein complex, inhibition of which blocks mouse oocytes in the GV stage or in anaphase I (Wang et al., 2004; Zhang et al., 2007). Reduced transcription of *Dync1i1* in aged oocytes could therefore be associated with impaired competence. *Ppp3ca* encodes calcineurin, which plays important roles in meiosis maturation in oocytes from many species (Zhang et al., 2019) and may contribute to regulation of GVBD in mouse oocytes (Wang et al., 2017). There were also changes in genes encoding activities potentially involved in RNA processing, such as the nuclear cyclophilin component PPIL3 (Rajiv & Davis, 2018), but whose relevance in oocytes has not yet been explored. It is possible that the heterogeneity in expression and methylation of these and other genes could contribute to variable developmental competence of the oocyte or predispose to variable phenotypes in offspring.

## 4 EXPERIMENTAL PROCEDURES

### 4.1 Oocyte collection

All experimental procedures were performed under licences issued by the Home Office (UK) in accordance with the Animals (Scientific Procedures) Act 1986 and were approved by the Animal Welfare and Ethical Review Board at the Babraham Institute.

Fully grown germinal vesicle (GV) oocytes were collected from the ovaries of spontaneously ovulating C57BL/6Babr virgin females aged 12 weeks (young) or 44‐54 weeks (old, as previously defined (Woods et al., 2017)). Oocytes were released from the ovaries mechanically with a fine needle and cleaned in M2 medium (Sigma). Clean oocytes were washed twice in PBS, inspected to ensure complete removal of adherent granulosa cells, then individually lysed and flash‐frozen in 5 μl RLT Plus buffer (Qiagen) and stored at −80°C until further use. A total of 42 young and 46 old oocytes were collected from three young and seven old mice for library preparation.

### 4.2 DNA and RNA isolation

DNA and RNA from individual oocytes were physically separated using the G&T protocol (Angermueller et al., 2016). Briefly, Smart‐seq2 oligo‐dTs (Picelli et al., 2013, 2014) were annealed to magnetic beads (MyOne C1, Life Technologies) and used to capture polyadenylated mRNA from individual oocyte lysates. The remaining lysate containing the DNA was transferred to a separate tube and the beads washed three times in 1xFSS buffer (Superscript II, Invitrogen), 10 mM DTT, 0.005% Tween‐20 (Sigma) and 0.4 U μl^−1^ of RNAsin (Promega). The washing solutions were added to the DNA tube to maximise recovery. mRNA on the beads was immediately processed further for cDNA conversion by resuspending beads in 10 μl of reverse transcriptase mastermix (100 U SuperScript II (Invitrogen), 10 U RNAsin (Promega), 1 × Superscript II First‐Strand Buffer, 5 mM DTT (Invitrogen), 1 M betaine (Sigma), 9 mM MgCl_2_ (Invitrogen), 1 μM Template‐Switching Oligo (TSO, Eurogentec (Picelli et al., 2013, 2014)), 1 mM dNTP mix (Roche)). The mRNA mixture was reverse transcribed by incubation for 60 min at 42 °C followed by 30 min at 50 °C and 10 min at 60 °C. The cDNA was amplified by PCR by adding 11 μl of 2 × KAPA HiFi HotStart ReadyMix and 1 μl ISPCR primer (2 μM; (Picelli et al., 2013, 2014)). Samples were incubated in a thermocycler at 98 °C for 3 min, followed by 18 cycles of 98 °C for 15 s, 67 °C for 20 s, 72 °C for 6 min and finally 72 °C for 5 min. The amplified product was purified using Ampure XP beads with a 1:1 ratio and eluted into 20 μl of water.

In parallel, the lysate containing the DNA was purified using a 0.8:1 volumetric ratio of Ampure XP Beads (Beckman Coulter) and eluted into 10 μl of water to use for scBS‐seq library preparation.

### 4.3 Single‐cell RNA‐sequencing

Libraries were prepared from 100 to 400 pg of cDNA using the Nextera XT Kit (Illumina), per the manufacturer's instructions but with one‐fifth volumes. All 88 single‐cell RNA‐seq libraries were pooled together and sequenced on the Illumina NextSeq platform to an average depth of 4.2 million reads (Table S1), using single‐end 100 bp read‐length settings.

### 4.4 Single‐cell BS‐sequencing

Single‐cell BS‐seq (scBS‐seq) libraries were prepared as previously described (Clark et al., 2017; Smallwood et al., 2014), with minor changes. DNA purified from single cells was bisulphite‐converted using the EZ Methylation Direct Kit (Zymo) according to the manufacturer's instructions, but using half volumes. Five rounds of first‐strand synthesis were carried out, starting by eluting bisulphite‐converted DNA into 40 μl of first‐strand synthesis mastermix (1 × Blue Buffer (Enzymatics), 0.4 mM dNTP mix (Roche), 0.4 μM 6NF oligo (IDT)). The mixture was heated to 65 °C for 2 min and cooled on ice. 50U of Klenow exo‐ (Enzymatics) was added, and the mixture incubated on a thermocycler at 37 °C for 30 min after slowly ramping from 4 °C. First‐strand synthesis was repeated 4 more times with the addition of 2.5 μl of reaction mixture (1× blue buffer, 0.25 mM dNTPs, 10 mM 6NF oligo and 25U Klenow exo‐). In the final round, samples were incubated for 90 min at 37 °C. Exonuclease digestion was carried out by adding 20U of exonuclease I (NEB) to the reactions, diluting with water to a total volume of 100 μl, followed by an incubation at 37 °C for 1 hour. Samples were purified with AMPure XP beads using a 0.75:1 ratio. Beads were resuspended in 50 μl second‐strand master mix (1× Blue Buffer (Enzymatics), 0.4 mM dNTP mix (Roche), 0.4 μM 6NF oligo (IDT). Reactions were heated for 98 °C for 1 min, cooled on ice, before adding 50U of Klenow exo‐ (Enzymatics). The mixture was incubated on a thermocycler at 37 °C for 90 min after slowly ramping from 4 °C. Samples were purified using a 0.75:1 ratio of AMPure XP beads, and libraries were amplified in 50 μl of PCR mastermix (1× KAPA HiFi Readymix, 0.2 μM PE1.0 primer, 0.2 μM iTAG index primer) using the following protocol: 2 min at 95 °C, 14 cycles of 80 s at 94 °C, 30 s at 65 °C, 30 s at 72 °C and final extension for 3 min at 72 °C. Finally, scBS‐seq libraries were purified using a 0.7:1 volumetric ratio of AMPure XP beads and eluted in 15 µl EB buffer before pooling and sequencing.

Pools of ~20 libraries were generated and sequenced on 4 lanes on the Illumina HiSeq 2500 platform. Libraries were sequenced to an average of 13.0 million paired‐end reads (Table S6) with 125 bp read‐length.

### 4.5 Data processing

#### 4.5.1 scRNA‐seq data processing

A total of 88 libraries were adapter and quality trimmed (Phred score <20) using Trim Galore version 0.4.4 (Krueger, 2017). Reads with a minimum length of 20 bp after trimming were retained for downstream processing. Trimmed sequences were aligned to the Genome Reference Consortium mouse genome build 38 (GRCm38) with HiSat2 version 2.1.0 (Kim et al., 2015) in single‐end mode using a high penalty score for soft‐clipping (‐‐sp 1000,1000) and ‐‐dta for alignment reporting. Primary alignments were used for gene expression quantification over features of the mouse oocyte transcriptome (Veselovska et al., 2015). One oocyte was identified as an outlier in a distance matrix (cut‐off =0.95‐quantile) and was excluded from downstream analyses.

#### 4.5.2 scBS‐seq data processing

A total of 80 scBS‐seq libraries (39 young and 41 aged oocytes) were processed for analysis. The first 6 bp containing the N portion of the random primers, adapters and bases called with poor quality (Phred score <20) were removed using Trim Galore version 0.4.4 (Krueger, 2017) in single‐end mode. Reads with a minimum length of 20 bp after trimming were retained for downstream processing. Bismark version 0.18.2 (Krueger & Andrews, 2011) was used for read alignment, deduplication and methylation calling. Alignment to GRCm38 was performed in single‐end non‐directional mode followed by deduplication (deduplicate_bismark) and methylation calling (bismark_methylation_extractor). scBS‐seq libraries having a mapping efficiency <10%, fewer than 500,000 CpGs covered, or greater than 50% methylated CpGs were discarded. An additional quality control we applied as a means of identifying possible somatic cell (granulosa cell) contamination that might have been missed by visual inspection was to assess the average methylation of X chromosome CpG islands, with the rationale that CpG islands on the inactive X chromosome in female somatic cells are highly methylated, whereas during oocyte development the X chromosomes have undergone reactivation. All oocyte libraries had average X chromosome CpG island methylation rates <10%, compared with sequenced single granulosa cells with X chromosome CpG methylation >20%. In total, 30 young and 32 aged oocytes were kept for downstream analyses. On average, 3,203,801 CpGs per oocyte were interrogated (Table S6).

### 4.6 Differential expression and differential variability

Expression levels were normalised using size factors, and differential expression was tested using the R package DESeq2 (Love et al., 2014) at a false discovery threshold (FDR) of 5%. Differential variability was tested using the R package BASiCS (Vallejos et al., 2015) with an expected false discovery rate of 10%.

### 4.7 Differential methylation analysis and cell clustering

In the fully grown GV oocyte, global DNA methylation is around 40% with methylation states shared along broad clusters of unmethylated and methylated CpG sites. Veselovska et al., 2015, identified and termed clusters as hypermethylated (75‐100% methylation) and hypomethylated (0‐25% methylation) domains with median sizes of 20.9 kbp and 24.9 kbp, respectively. Here, DNA methylation was quantified over these distinctive domains and the interdomains between these two in random groupings of 10 oocytes (pseudobulk groups). Differential methylation was tested by fitting a weighted logistic regression model. To limit the rate of false‐positive signals due to the composition of the groups, one hundred different combinations of oocytes belonging to the two groups were computed. Signals called significant at a false discovery rate of 5% in at least 95% of the combinations were called age‐associated differentially methylated regions. Single oocytes were clustered into two groups based on their patterns of DNA methylation using Melissa (Kapourani & Sanguinetti, 2019), a Bayesian method for imputation and clustering of single‐cell methylomes.

### 4.8 Gene expression and DNA methylation pairwise correlations

Pearson's correlation was used to identify linear relationships between DNA methylation and gene expression levels. For this analysis, DNA methylation was quantified at gene bodies over single oocytes.

## CONFLICT OF INTEREST

The authors declare no competing interests.

## AUTHOR CONTRIBUTIONS

MH, GK conceived and supervised the study; EH‐P generated sequencing data sets with assistance from SJC; JC‐F performed bioinformatic analysis with input from HD, CH; JC‐F, HD, EH‐P, GK wrote the manuscript with input from all other authors.

## Supporting information

Figure S1‐S5

Table S1‐S7

## Data Availability

Sequence data that support the findings of this study have been deposited under accession code GSE154370 in the Gene Expression Omnibus database at https://www.ncbi.nlm.nih.gov/geo/query/acc.cgi?acc=GSE154370.
