# Correction to ‘Increased transcriptome variation and localised DNA methylation changes in oocytes from aged mice revealed by parallel single‐cell analysis’

**DOI:** 10.1111/acel.14364

**Published:** 2024-10-29

Castillo‐Fernandez, J., Herrera‐Puerta, E., Demond, H., Clark, S.J., Hanna, C.W., Hemberger, M. and Kelsey, G. (2020), Increased transcriptome variation and localised DNA methylation changes in oocytes from aged mice revealed by parallel single‐cell analysis. *Aging Cell*, 19: e13278. https://doi.org/10.1111/acel.13278.

In the assignment of individual GV oocytes as having NSN or SN chromatin configuration based on scRNA‐Seq profiles, we used a published gene list, in which we now believe NSN and SN samples may have been mis‐assigned. We have now generated our own scRNA‐Seq datasets of NSN and SN oocytes (https://doi.org/10.21203/rs.3.rs‐4901993/v1), which now enables us to correctly reassign NSN and SN status of GV oocytes in this paper. This necessitates the following corrections:

In Figure 1, the labelling of NSN and SN in panels (c), (d) and (e) was incorrect. In the legend, “triangles” and “circles” have been transposed and “grey” and “black” text was incorrect. The text “MII oocyte” should read “GV oocyte.” The labelling of NSN and SN was incorrect as well in panel (d). The correct figure and legend are shown below.

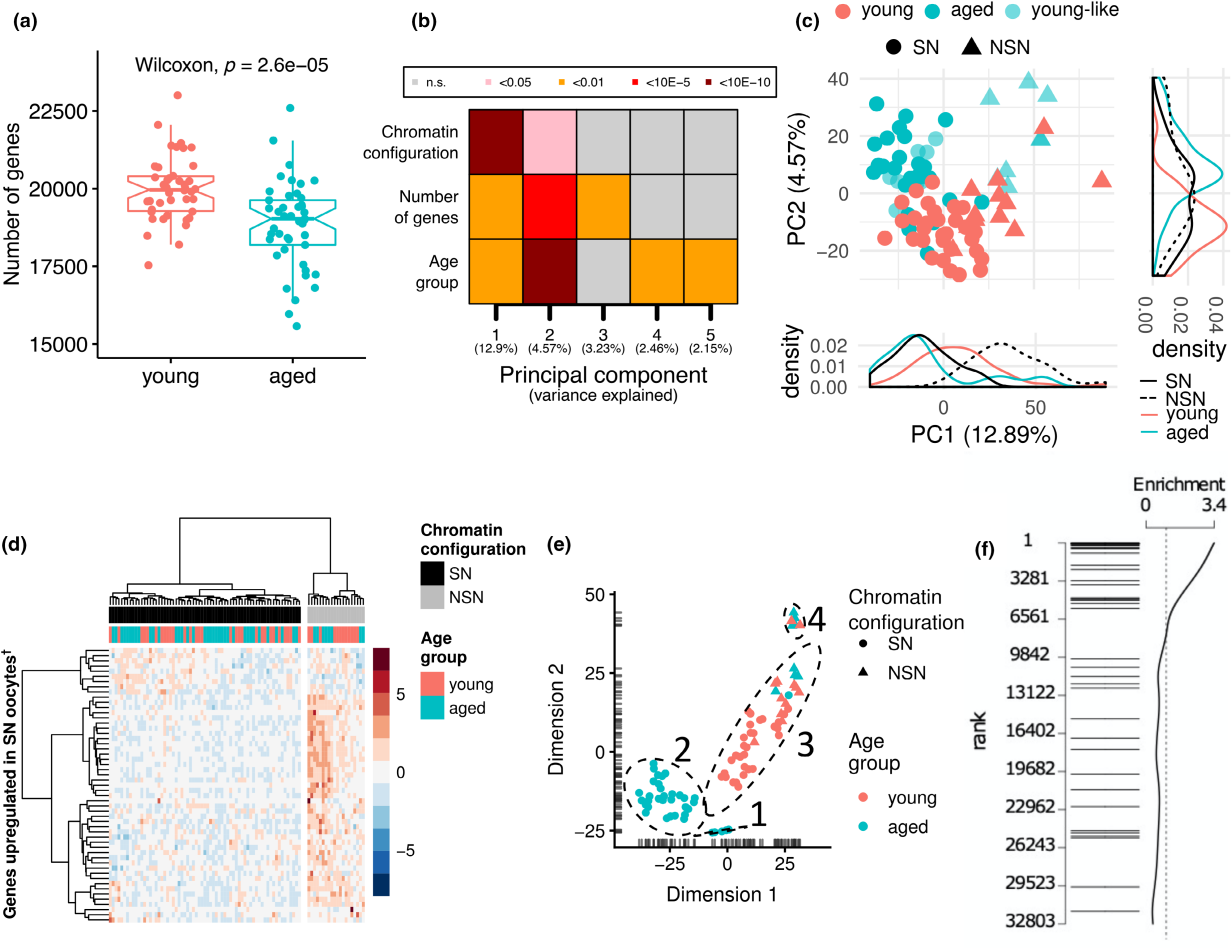

FIGURE 1 Transcriptomic profiles of oocytes from young and old females. (a) Boxplots of transcript diversity showing a decrease in oocytes from aged mice (Wilcoxon test; *p* = 2.6 x 10^‐5^). Each dot represents the number of genes detected at ≥1 counts in scRNA‐seq of an individual GV oocyte. (b) Heatmap of the associations (linear regression) between a transcriptional signature of chromatin configuration, number of detected transcripts and age with the first five principal components of the transcriptome. (c) Principal component analysis (PCA) plot of the transcriptome of oocytes from young and aged mice. Principal component 1 (PC1) is highly explained by a predicted chromatin configuration (non‐surrounded nucleolus (NSN): triangles; surrounded nucleolus (SN): circles). PC2 is highly explained by age (young: red; aged: turquoise). A subpopulation of aged oocytes with young‐like (light turquoise) features is also demarcated. (d) Hierarchical clustering for the prediction of chromatin states of oocytes as NSN (grey) or SN (black) according to the level of expression of genes overexpressed in NSN oocytes. (e) t‐SNE plot showing four main clusters of oocytes driven by inferred chromatin state and/or age group. (f) Barcode plot showing the enrichment of maternal effect genes when testing for differences across the four main clusters of oocytes. Each horizontal bar represents one maternal effect gene. The position of the bar along the y‐axis represents its ranking across all expressed genes tested for differential expression (1 being the most significant)

In Figure 2, the labelling of NSN and SN in panel (e) was incorrect. The correct figure and legend are shown below.

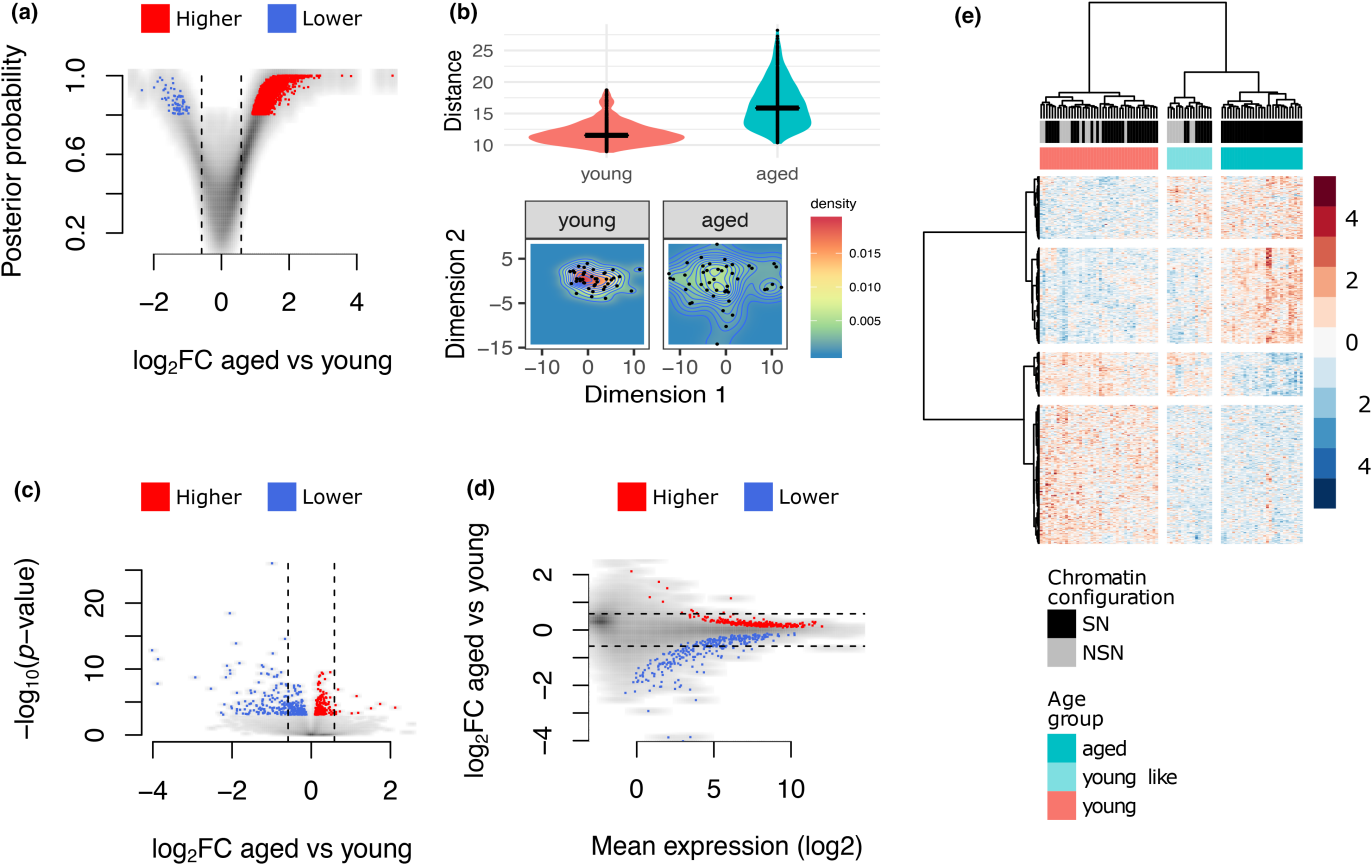

FIGURE 2 Differences in variability and mean levels of transcript abundance. (a) Volcano plot of differential variability between young and aged oocytes showing a greater number of genes having more variable expression values in the aged group. (b) Upper: boxplot of the pairwise distances between cells of the same age group. Lower: heatmap of the 2D distribution of oocytes using the expression level of genes identified as differentially variable between young and aged oocytes. (c) Volcano plot of differential mean expression between young and aged oocytes. (d) Scatter plot of the effect size vs. mean expression level of genes tested for differential expression. (e) Hierarchical clustering of oocytes using expression levels of 560 age‐associated genes showing a young‐like subgroup within the aged oocytes. In a, c, d, red and blue dots represent higher or lower variability (a) and mean expression (c, d), respectively, in the aged group

In Figure 3, the labelling of NSN and SN in panels (b), (d) and (e) was incorrect. The figure has been replaced with a corrected version in which NSN and SN labelling in panels (b), (d) and (e) is corrected. The correct figure and legend are shown below.

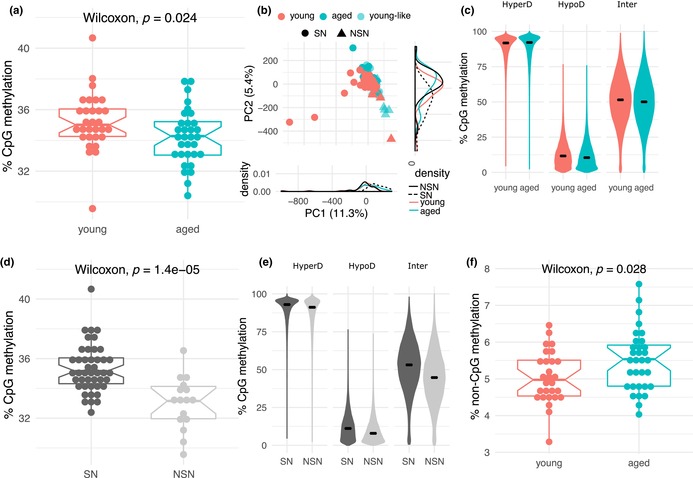

FIGURE 3 DNA methylation profiles of individual oocytes from young and aged females. (a) Boxplot of average CpG methylation values showing lower levels in aged oocytes (Wilcoxon test; *p* = 0.024). Each dot represents the global CpG methylation estimate from scBS‐seq of 32 individual GV oocytes from young and 30 from aged females. (b) PCA plot of oocyte CpG methylomes of young and aged oocytes based on the average methylation at hyper‐, hypo‐ and intermediately methylated domains. (c) Violin plots of the distribution of average CpG methylation values across three types of methylation domains in the oocyte: hypermethylated (HyperD: 75‐100%); hypomethylated (HypoD: 0‐25%) and intermediately methylated (Inter: 25‐75%) in young and aged oocytes. scBS‐seq data merged by group. (d) Boxplot of average CpG methylation values in oocytes assigned as NSN and SN (Wilcoxon test; *p* = 1.4x10^‐5^). (e) Violin plots of the distribution of average CpG methylation values of HyperD, HypoD and Inter domains in oocytes assigned as NSN and SN. scBS‐seq data merged by group. (f) Boxplot of average non‐CpG methylation values in young and aged oocytes (Wilcoxon test; *p* = 0.028)

In the Results sections 2.1, 2.2, 2.4, 2.5: the terms NSN and SN were used incorrectly as a result of the mis‐assignment. The corrected sentences are provided below:

**Section 2.1**

“To assign chromatin configuration states in our data set, we classified the 87 oocytes according to the level of expression of genes reported to show at least a two‐fold overexpression in NSN oocytes compared to SN oocytes (data reanalysed from Ma et al., 2013). Twenty oocytes were found to express these transcripts at a higher level and were classified transcriptionally as NSN (Figure 1d).”

“Out of the 20 NSN oocytes, twelve corresponded to the young group and eight to the aged one.”

**Section 2.2**

“Clusters 1 and 2 comprised only SN aged oocytes; Cluster 3 mainly comprised young SN oocytes plus a small number of NSN oocytes; and Cluster 4 purely NSN oocytes regardless of age, which were closer to young SN oocytes (Cluster 3) than to aged SN oocytes (Clusters 1 and 2).”

“Following this assumption, differential expression was tested using cluster number as a continuous variable to identify transcripts that change in abundance from old transcriptionally like SN oocytes to young transcriptionally like SN oocytes and, lastly, to NSN oocytes (both young and aged).”

“To exclude that the observed differences in transcript abundance of maternal effect genes were an effect solely of chromatin configuration, the analysis was repeated using only the subset of oocytes assigned as SN and the enrichment for maternal effect genes was also observed (Figure S1).”

**Section 2.4**

“Interestingly, all of the predicted NSN aged oocytes were also defined as young‐like oocytes, although the young‐like group also contained some aged oocytes assigned as SN (Figure 2e).”

**Section 2.5**

“However, when examining differences between assigned chromatin configurations, lower CpG methylation was observed in NSN‐classified oocytes both globally (Wilcoxon test; *p* = 1.4 × 10^−5^) and in all three categories of genomic features (Figure 3d,e).”

The following statement in Section 2.1 should be disregarded, “However, we also observed that the number of SN oocytes was low compared to the numbers reported in the literature, especially in our aged group in which more than 80% of oocytes are expected to be SN. It is likely that the classification obtained using gene expression as a proxy does not reflect the actual chromatin state, but instead suggests that most aged oocytes expected to be SN express an immature NSN‐like transcriptome.”

In Section 2.2, “Cluster 1” and “Cluster 4” should be transposed in the following sentence: “This spatial relationship suggested a trajectory from an immature NSN transcriptome (Cluster 4) to a mature SN one (Cluster 1).”

The following statement should be added to the Methods in section 4.6: “NSN and SN status of GV oocytes was inferred from the scRNA‐seq data using a list of genes identified as differentially expressed in NSN versus SN oocytes from Ma et al. (2013). Note, however, that we believe the NSN and SN samples in Ma et al. (2013) were mis‐assigned, such that genes overexpressed in SN oocytes should refer to genes overexpressed in NSN oocytes (https://doi.org/10.21203/rs.3.rs‐4901993/v1).”

The following wording in the Discussion is no longer correct and should be disregarded: “We scored a surprisingly high proportion of oocytes from older females as NSN from their transcriptomes at an age when the ovary should contain few NSN oocytes (Zuccotti et al., 1995). On the other hand, it has been reported that more than a quarter of GV oocytes in old female mice cannot be classified as either NSN or SN, but display anomalous chromatin configurations (Manosalva & González, 2010). Together, these observations suggest that aged oocytes may have undergone the NSN‐SN transition, but imperfectly and without the full transcriptome maturation that ensures developmental competence.”

The reassignment of the inferred NSN and SN status of oocytes does not otherwise alter the findings of the paper.

We apologize for these errors.

