# Supplementary material for: Increased transcriptome variation and localised DNA methylation changes in oocytes from aged mice revealed by parallel single‐cell analysis
Source: Aging Cell. 2020 Nov 17;19(12):e13278. doi: 10.1111/acel.13278 (PMC7744954; doi:10.1111/acel.13278)
Supplement: Supplementary file 1 — Figure S1‐S5 [file ACEL-19-e13278-s001.docx]

**Increased transcriptome variation and localised DNA methylation changes in oocytes from aged mice revealed by parallel single-cell analysis**

Juan Castillo-Fernandez, Erika Herrera-Puerta, Hannah Demond, Stephen J. Clark, Courtney W. Hanna, Myriam Hemberger, Gavin Kelsey

**Supplementary Figures**


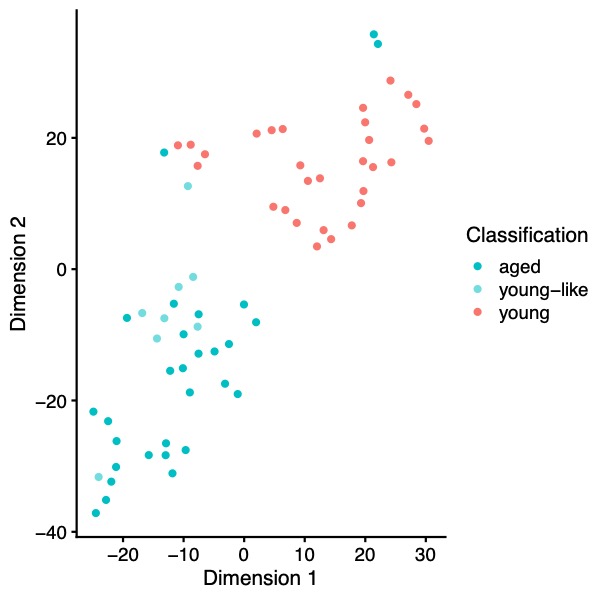


**Figure S1. Clustering of transcriptomes of oocytes assigned as NSN-like.** Unsupervised clustering of transcriptomes of oocytes, young (n=30) and aged (n=37), is driven by age and not exclusively by chromatin configuration as shown by t-SNE plot.

**Figure S2. Principal components of methylomes associated with age.** Boxplots of the scores of young (n=30) and aged (n=32) oocytes along principal components (PCs) 1 and 2. Principal components analysis of methylomes of oocytes was based on methylation levels at hyper-, hypo- and intermediately methylated domains.

**Figure S3. Imprinted gDMR methylation at single-cell level.** Heatmap showing methylation levels at 20 maternal gDMRs, three paternal gDMRs (*Rasgrf1, Dlk1-Gtl2, H19_Igf2*) and two secondary DMRs (*Nesp* and *Gpr1-Zdbf2*). Each dot represents a single oocyte, young (n=30) or aged (n=32) as indicated by the bottom labels (red: young, blue: aged), at the locus indicated by the *y*-axis. The size of the dot represents the read count for that oocyte at the specific DMR. The dot colour represents the percentage of methylation. Low variability is observed across cells for the same DMR.

**
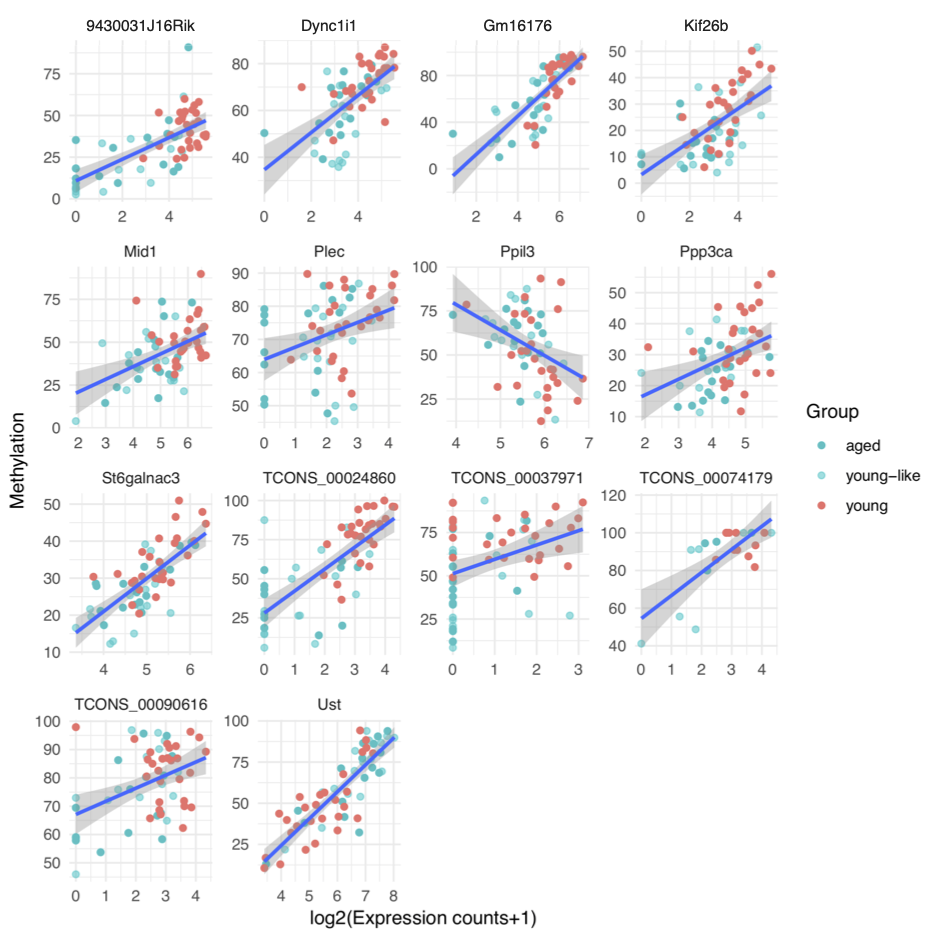
**

**Figure S4. Coordinate changes in gene expression and CpG methylation.** Scatter plot of 14 genes in which a significant Pearson’s correlation (p<0.05) was observed between gene-body methylation and gene expression at single-cell level using oocytes from young (n=30) and aged (n=32) mice.

**Figure S5. Non-CpG methylation is positively correlated with CpG methylation at gene bodies.** Scatter plot of non-CpG vs CpG methylation at 23 genes with identified age-related changes in both CpG methylation and expression at single-cell level in oocytes from young and aged mice (n=8-62). A significant positive correlation (Pearson’s correlation) (p<0.05) was observed for these genes, except for a single, oocyte-specific non-coding transcription unit (TCONS_00074179).
